# The diel pattern in harbour porpoise clicking behaviour is not a response to prey activity

**DOI:** 10.1038/s41598-020-71957-0

**Published:** 2020-09-10

**Authors:** Anna N. Osiecka, Owen Jones, Magnus Wahlberg

**Affiliations:** 1 Marine Biological Research Centre, Department of Biology, University of Southern Denmark, Hindsholmvej 11, 5300 Kerteminde, Denmark; 2 Department of Biology, University of Southern Denmark, Campusvej 55, 5230 Odense, Denmark; 3 Interdisciplinary Centre on Population Dynamics (CPOP), University of Southern Denmark, Campusvej 55, 5230 Odense, Denmark

**Keywords:** Zoology, Animal behaviour

## Abstract

Wild harbour porpoises (*Phocoena phocoena*) mainly forage during the night and, because they rely on echolocation to detect their prey, this is also when they are most acoustically active. It has been hypothesised that this activity pattern is a response to the diel behaviour of their major prey species. To test this hypothesis, we monitored the acoustic activity of two captive harbour porpoises held in a net pen continuously during a full year and fed by their human keepers during daylight hours, thus removing the influence of prey activity. The porpoises were exposed to similar temperature and ambient light conditions as free-ranging animals living in the same region. Throughout the year, there was a pronounced diel pattern in acoustic activity of the porpoises, with significantly greater activity at night, and a clear peak around sunrise and sunset throughout the year. Clicking activity was not dependent on lunar illumination or water level. Because the porpoises in the pen are fed and trained during daylight hours, the results indicate that factors other than fish behaviour are strongly influencing the diel clicking behaviour pattern of the species.

## Introduction

Acoustic communication and echolocation are of extreme importance for harbour porpoises (*Phocoena phocoena*) and are used as means of finding food, navigation, and communication^1–4^. Porpoises only produce one type of acoustic signal, a very high frequency (130 kHz) and short duration (50 µs) click^5^. By varying the repetition rate of clicks they can either use them for echolocation^6^ or communication^3^. Clicks are produced throughout the day and night, and year round^7,8^. Harbour porpoises have acute hearing abilities already fully developed in neonate animals^9^ and one of the lowest hearing thresholds found in any animal^10^, indicating the importance of acoustic cues for this species.

The porpoise stereotypical and easily recognisable signal makes this species extremely suitable for passive acoustic monitoring (PAM) to provide information on their presence in time and space, as well as on their bioacoustic behaviour^11–13^. Porpoises emit signals in a very narrow beam^14,15^, which decreases the likelihood of detecting the signals. However, the fact that clicks are produced almost continuously and that animals are constantly moving around has made PAM a very important tool for studying this species in the field. It is also widely used to successfully assess the impact on porpoises from many man-made activities, such as noise from coastal wind farms^1,16–19^ and deterrent signals intended to keep them away from gill nets^20–24^.

From PAM studies, distinct diel rhythms in harbour porpoise acoustic activity have been described in the inner Danish and Swedish west coast waters^8,25,26^, and the North Sea^27,28^, with a strong peak in echolocation activity around midnight and considerably lower activity during the day. An even more pronounced nocturnal peak was observed in the proximity of industrial in-water structures, such as bridge pillars^29^. The increased night-time clicking activity might be attributed to the loss of visual information at night, diel movement patterns of prey, or intrinsic physiological diurnal phases and/or lunar cycles^25^. Lunar (and thus, in some waters, tidal) cycles, as well as light (both solar and lunar), are of great importance triggering behaviours such as vertical migrations and schooling patterns in many species of fish that are preyed upon by porpoises^30–34^.

Here, the acoustic activity patterns of captive harbour porpoises are investigated over a full year to understand which extrinsic cues are triggering their daily clicking behaviour. The porpoises are kept in a net pen under ambient water conditions and fed regularly at specific times during daylight. Therefore, when compared to wild porpoises, cues generated by prey behaviour can be ruled out, but a strong additional cue, feeding events in the daytime, has been introduced. The acoustic activity analysis investigates whether the porpoises have a steady diel clicking pattern also in the net pen, and whether factors other than fish behaviour are influencing the acoustic activity patterns of this species.

## Methods

### Location and study subjects

Recordings were made 15 m from the porpoise pen of Fjord&Bælt facility in Kerteminde, Denmark. At the time of the study, this facility kept two adult female harbour porpoises in captivity for research and educational purposes. Both animals were wild-born and brought to the facility after getting accidentally caught in demersal nets in 1997 (porpoise named Freja) and 2004 (porpoise named Sif). The porpoises are kept in a 30 × 20 m pen with large-meshed nets keeping the animals in ambient water conditions year round. The animals receive food during several daily training sessions using positive reinforcement. Specialised caretakers regularly monitor their health, behaviour, and general well-being. The animals are held at the Fjord&Bælt under permit J.nr. SVANA-610-00084, Ministry of Environment and Food, Denmark. All methods were carried out in accordance with relevant guidelines and regulations. This study did not directly involve the animals and used passive monitoring instead, thus no additional permits were required.

### Data collection

**Figure 1:**
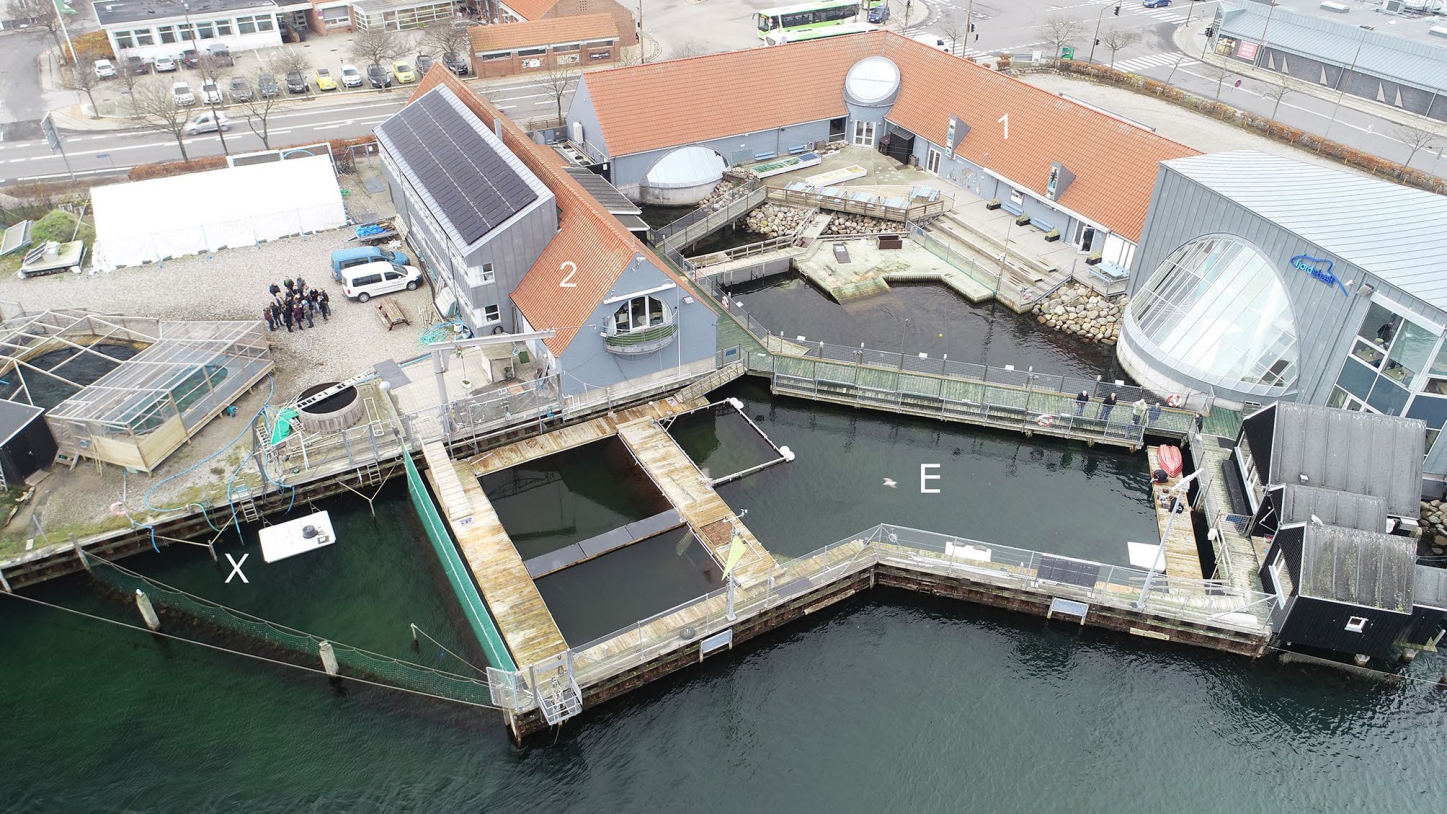
Deployment set-up. E: porpoise enclosure at Fjord & Bælt; 1: Fjord & Bælt facility; 2: Marine Biological Research Centre; X: C-POD deployment site. Photo by Sara Ortiz.

A C-POD data logger (Chelonia, Inc.) was anchored in Kerteminde harbour, 15 m from one end of Fjord&Bælt’s porpoise pool (Fig. 1). The C-POD was calibrated both before and after the deployments using standard methods^35^ to confirm that the equipment functioned properly and to establish its detection threshold for porpoise clicks (115 dB re 1 µPa rms).

The logger was anchored at 3 m depth, 1 m above the bottom, and more than 1 m away from the harbour wall. It was located 15 m from the closest part of the harbour porpoise enclosure. The C-POD monitored porpoise acoustic activity in the period from the 6th of June 2016 to the 7th of June 2017. The equipment was retrieved eight times to download the collected data, change batteries, and ensure the gear was still running correctly, remaining on land for 20–60 min each time before being deployed again.

Data collected from 12:00 on the 6th of June 2016 to 11:59 on the 6th of June 2017 were selected for analysis. During the entire 365 days of recordings, there were in all 7 days with no collected data due to technical issues with the data logger and data retrieval. Only the periods with no missing data were included in the analysis, further limiting the sample size to 345 for daylight and 354 for night-time.

### Porpoise data analysis

The acoustic data were analysed using *CPOD.exe.* software V2.044 (Chelonia Ltd., Mousehole, UK). Porpoise click trains were identified using the KERNO classifier with “Hi” quality (the highest level of probability) and “NBHF” (narrow band high frequency) filters. Data were exported as the number of clicks detected per minute^36,37^. Data from 5 min after each deployment and from 2 min before each retrieval of the equipment were removed to exclude data before the logger had stabilised itself in the water as well as any immediate curious biosonar inspection by the animals to the logger’s presence.

### Data on abiotic factors

R package *lunar*^38^ was used to obtain the lunar phase and illumination data and R package *suncalc*^39^ to obtain sunrise and sunset times. Data on sea surface height during the recording period were obtained from the Danish Coastal Authority’s measurement station in Kerteminde harbour less than 50 m from the porpoise facility at Fjord&Bælt.

### Statistical analysis

First, median clicks emitted per minute was calculated for each period of darkness (i.e. between sunset and sunrise) and each period of light (i.e. between sunrise and sunset). This resulted in a total of 699 measures (345 for daylight and 354 for night-time—the difference between these sample sizes is caused by short periods of incomplete data collection mentioned above).

Randomisation tests were used to determine whether the recorded number of clicks per minute differed between daytime and night-time, and between the crepuscular hours (defined here as 2 h before sunrise and after sunset) and other times. The same approach was used to determine whether there is an association between night-time click activity and (a) lunar phase and (b) water level. To conduct the tests, each night was assigned to one of the lunar phases: new, waxing, full, and waning. Lunar illumination was discretised into equally spaced groups of 0.00–0.25, 0.25–0.5, 0.5–0.75 and 0.75–1.00. Sea surface height was discretised into groups of low tide (− 59–28 cm) and high tide (28–115 cm).

Randomisation tests are a class of distribution-free method that ask where an observed test statistic (in this case the difference in average (median) number of clicks emitted per minute between two groups) falls in relation to a null distribution that is generated by randomising the explanatory variable in the data at hand many times^40^. If the variable has little explanatory power, then randomising it would have very little effect on the test statistic. A p-value can thus be calculated as the proportion of replicates where the randomised test statistic is greater than or equal to the absolute value of the observed test statistic. A major advantage of using randomisation tests is that they do not violate the assumptions of ordinary linear models such as normality, homogeneity of variances, and independence of errors. 5,000 replicates were used for all randomisation tests.

The time of the highest concentration of clicks and its 95% confidence interval were obtained from the maximum likelihood estimate of the parameters of a circular normal (von Mises) distribution using the *mle.vonmises* function from package *circular*^41^. In addition to the randomisation test described above, a Rayleigh test was used to test the significance of departure from a uniform (non-directional) distribution of click intensity^42^. All analyses were performed using R 3.5.2^43^.

## Results

### The influence of abiotic factors on porpoise clicking activity

**Figure 2:**
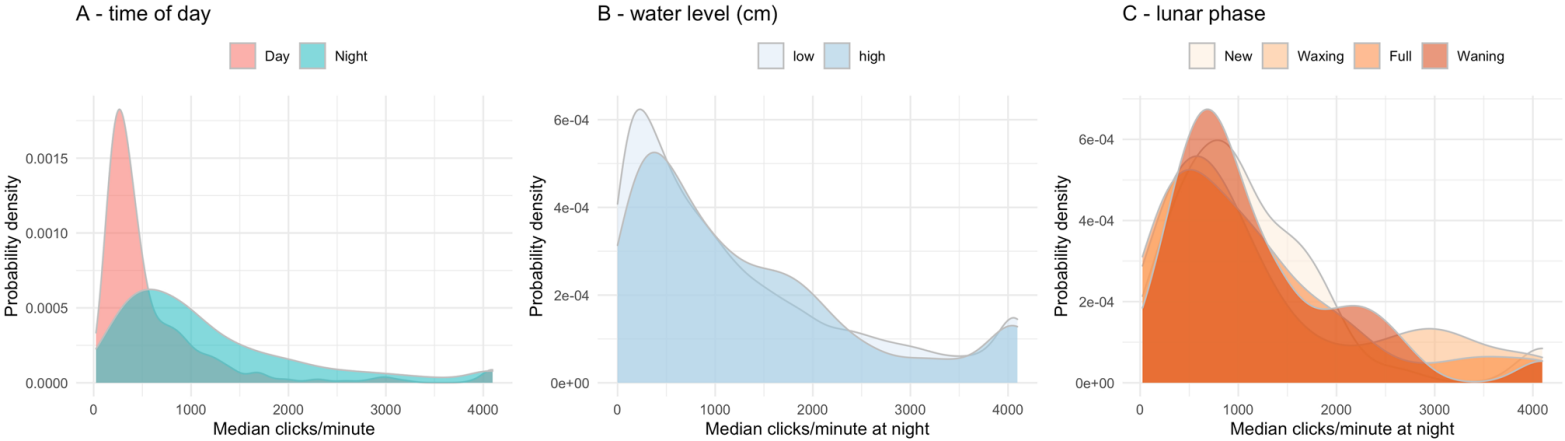
The effect of (**A**) time of day, (**B**) water level, and (**C**) nocturnal lunar phase on the number of porpoise clicks recorded per minute. The polygons show the probability density distributions of clicking activity in the (discretised) levels of these three abiotic factors. The difference between day and night-time was highly significant (two-tailed randomisation test: n = 5,000, p < 0.01). Clicking activity decreased very significantly when the water level was deeper (between 57 and 115 cm) (two-tailed randomisation test: n = 5,000, p < 0.01). Lunar phase had no significant effect on clicking intensity (two-tailed randomisation test: n = 5,000, p-values ranged between 0.21 and 0.49 in each of the pairwise comparisons).

**Figure 3:**
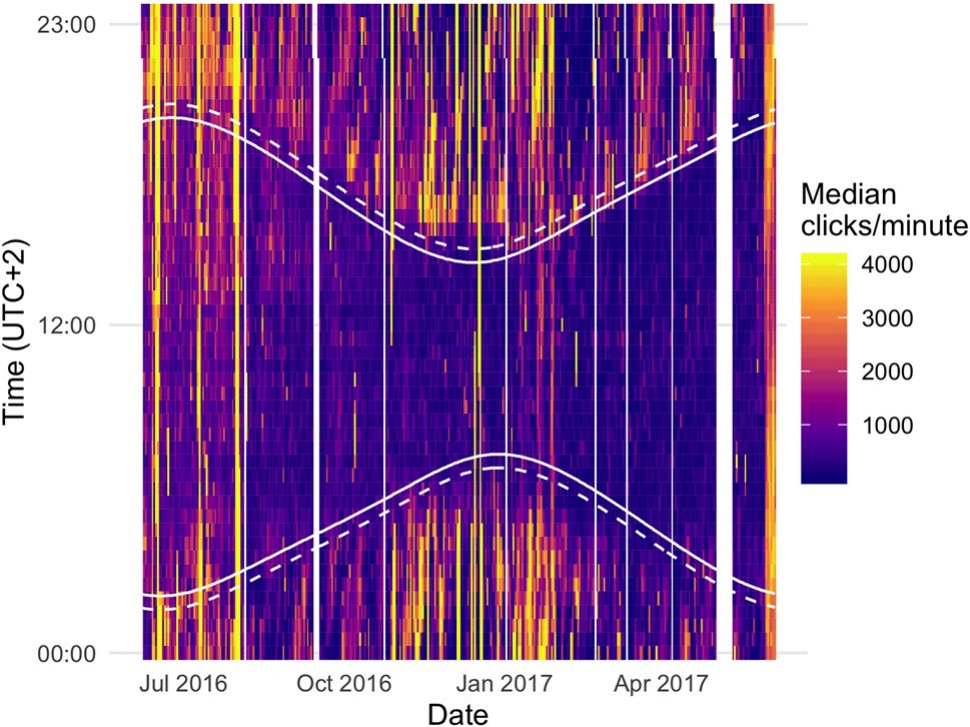
Number of clicks received per minute as a function of date and time, showing that highest number of clicks are detected during the night, while the daylight hours are relatively quiet. Number of clicks is expressed as median clicks per minute within each hour and is indicated by colour such that high intensities are yellow and low intensities are purple. The unbroken white lines indicate sunrise and sunset times while the broken white lines indicate 1 h before sunrise and 1 h after sunset. The vertical white strips indicate periods of missing data (e.g. where the data logger is out of the water).

**Figure 4:**
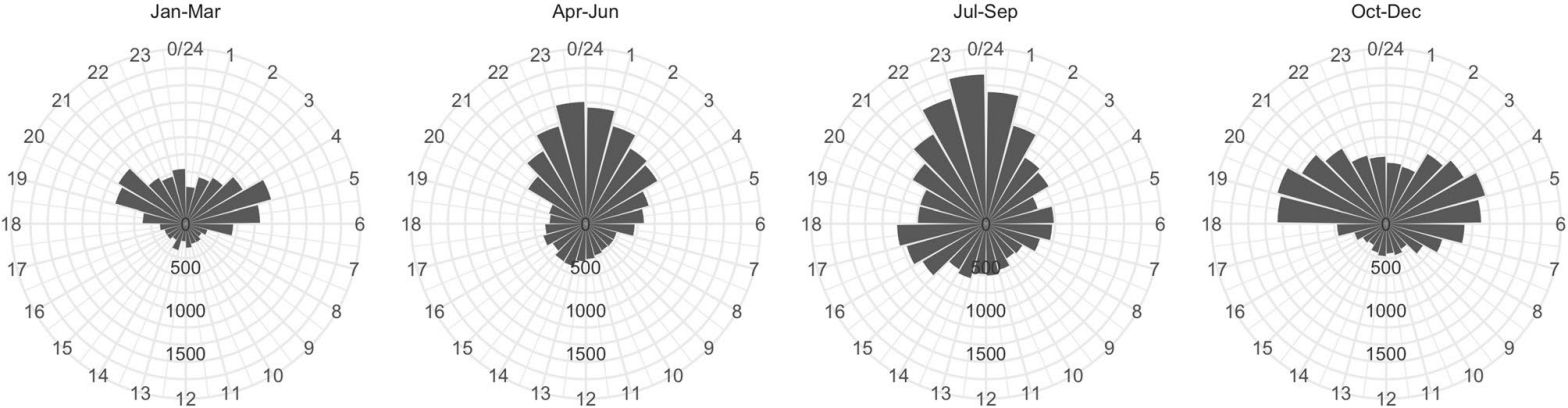
Median clicks per minute within each hour of a 24-h day during the four seasons. The length of the bars indicates the median clicks/minute and the angle of the bar represents the time (hour of day), indicated by the numbers at the outer circumference. The midnight peak in activity appears only during the summer months (April–September).

The average clicking activity was considerably higher during the night than during daylight hours throughout the year (Figs. 2A and 3). This difference was highly significant (two-tailed randomisation test: n = 5,000, p < 0.01). Clicking activity was found to be significantly higher in the crepuscular hours than at other times (two-tailed randomisation test: n = 5,000, p < 0.01). The Rayleigh test also indicated a highly significant departure from a uniform circular distribution of click intensity (test statistic = 0.262, p-value < 0.01). The overall peak-click time was estimated to be at 23:59 UTC + 2 (95% CI 23:50–00:09; Fig. 4). This midnight peak appeared to be present only during the summer months (April–September; Fig. 4), but this pattern is caused by the merging of the pre-dawn and post-sunset peaks that occur throughout the year.

Water level ranged from extremes of − 59 cm to 115 cm relative to the local average sea level, but the variation was normally much more modest (1st and 3rd quartiles were − 12 and 15 cm, respectively). There was no significant difference in average clicking activity between the high and low water level groups (Fig. 2B; two-tailed randomisation test: n = 5,000, p = 0.130). Lunar phase also had no significant effect on clicking activity (Fig. 2C; two-tailed randomisation tests: n = 5,000, p-values for the pairwise comparisons ranged between 0.210 and 0.490).

The highest clicking activity was observed in June and July (Fig. 3). Other than this, porpoise clicking activity gradually rose as light availability decreased over the autumn and winter months.

## Discussion

A strong diel clicking pattern with higher night-time activity and a strong midnight peak has often been reported for wild harbour porpoises in many different areas^25–28,44^. However, field-collected data cannot determine whether the porpoises increased their general clicking activity, whether the animals were simply more likely to visit the vicinity of the data logger, or whether they clicked with a higher intensity over certain periods. In this study, two of these factors could be ruled out, as both porpoises were kept in the vicinity of the data logger throughout the study year, and as the data logger was sufficiently sensitive to detect porpoise clicks over their range of produced sound levels. This study clearly indicates that captive harbour porpoises fed during daylight hours have a similar diurnal clicking pattern to wild porpoises, with most activity occurring during night-time hours. The overall increase in night-time acoustic activity and a summertime midnight peak confirmed in this study makes it very likely that wild porpoises exhibit their night-time peak due to increased clicking activity. As this pattern is found in both wild and captive animals, it further suggests another factor determining this behaviour than their foraging behaviour, such as porpoises having an intrinsic circadian rhythm or supplementing visual information in poor light conditions by echolocating.

Interestingly, a clear peak in clicking activity can be seen around sunset and sunrise throughout the year, with a “rest period” of a lowered night-time activity during the winter months. The activity peak is particularly strong during the summer months, so that, when all year’s data are pooled together, the overall activity pattern can be obscured by the summertime peak. Therefore, this study indicates that the midnight peak in clicking activity observed from studies in the wild is either a result of shorter monitoring times performed during the more accessible summertime months, or reflects an annual pattern obscured by the especially high summertime activity. The details of the clicking activity pattern of wild porpoises call for further investigation.

The highest average clicking activity in this study was observed in some of the months with the longest days (June and July). This was the case also outside of the opening hours at the facility and is therefore not the result of the tourist high season at the facility. Even though the acoustic activity gradually rose with the decreasing light availability from the beginning of autumn until January, it did not reach the activity levels observed in June and July. The high clicking levels in the summertime coincide with the harbour porpoise mating season^45^. It has been shown previously that harbour porpoises produce frequent social calls^4^ and can convey precise behavioural information by modifying repetition rate patterns of their clicks^3^. Their mating vocalisations, if such exist, have never been described. While it is not clear whether the peak activity times observed here are related to the mating season, it is possible that they might be a result of the hormonal status of the two captive females. Since the individuals studied here live in partial seclusion far away from any observed wild animals, and taking into account the high directionality and rather small active space^3^ of the sounds produced by these animals, it can be ruled out that vocalisations of wild porpoises, e.g. males attempting to mate, contributed to the observed peak. In any case, these observations open a door to further studies of harbour porpoise social behaviour. Furthermore, the higher clicking activity in the summertime cannot be explained by porpoises only regulating the sonic output to supplement the lack of visual information.

Stedt et al.^25^ had shown a significant effect of lunar phase on the acoustic activity of wild porpoises, with higher activity recorded at full moon than at new and quarter moon. In the same study, they detected no effect of water level on porpoise activity. In our study, there was neither an effect of lunar phase nor of water level. A strong lunar and tidal effect on clicking activity was previously reported for spinner, dusky and common dolphins^34,46^ and short-finned pilot whales^47^. It is likely that the correlation between sound production and lunar phases is due to prey activity patterns, as many species of fish and squid adjust their behaviour according to both tide and moon cycles. In some areas, there is a strong relationship between lunar phases and tides, and it is therefore difficult to separate how they affect both predators and prey. In Danish waters, however, water levels are only to a small extent determined by lunar phases and to a much larger degree by prevailing wind conditions. The fact that wild porpoises in inner Danish waters are affected by lunar cycles but not by water level strongly indicates that their prey is also more affected by moon phases rather than water height, which is not surprising due to the erratic pattern of water level changes in these waters. Moon cycles are also connected to varying lunar illumination, which can explain the higher or lower acoustic activity of an echolocating animal during different lunar phases. The fact that the activity pattern of captive porpoises seems unaffected by lunar phase may therefore be explained in two ways. Firstly, the patterns of wild porpoises could indicate that they do indeed respond to the behaviour of the prey species that depend on moon cycles but that are absent in captive conditions. However, as there seems to be no effect of lunar phases on the amount of produced feeding buzzes of wild porpoises^25^, it is possible that lunar illumination simply improves the ability of porpoises to use visual cues as a complement to echolocation. This could explain that the lunar phases are less obvious in the porpoise pen than in the open coastal waters, due to artificial street and building lights adjacent to the pen, providing night-time light sources dominating over moon light.

Caution needs to be taken interpreting behavioural data from captive animals. Perhaps the largest issue that needs to be taken into account in this study is the presence of training sessions several times a day. Even though porpoises in the pen of Fjord&Bælt do catch and search for wild fish entering their enclosure, they obtain a major part of their diet from the trainers during 3–5 daily feeding sessions spaced between 9.00 and 15:30. The fact that the Fjord&Bælt porpoises have a similar diel pattern as wild ones indicates that their diel activity rhythm is not dictated by the behaviour of their prey, but rather a response to the changing light availability. The presence of a night-time “rest period” during the winter and a pronounced crepuscular peak in acoustic activity of both wild and captive porpoises may indicate an intrinsic physiological rhythm or an undescribed behavioural pattern maintained in captive animals that is not caused by external stimulation and that calls for further investigation.

## Data Availability

The datasets generated during the current study are available from the corresponding author on reasonable request.

## References

[1] Verfuss UK, Sparling CE, Arnot C, Judd A, Coyle M, Popper AN, Hawkins A (2016). Review of offshore wind farm impact monitoring and mitigation with regard to marine mammals. The Effects of Noise on Aquatic Life II.

[2] Villadsgaard A, Wahlberg M, Tougaard J (2007). Echolocation signals of wild harbour porpoises, *Phocoena phocoena*. J. Exp. Biol..

[3] Clausen KT, Wahlberg M, Beedholm K, Deruiter S, Madsen PT (2011). Click communication in harbour porpoises *Phocoena phocoena*. Bioacoustics.

[4] Sørensen PM (2018). Click communication in wild harbour porpoises (*Phocoena phocoena*). Sci. Rep..

[5] Møhl B, Andersen S (1973). Echolocation: high-frequency component in the click of the harbour porpoise (*Phocoena* Ph. L.). J. Acoust. Soc. Am..

[6] Wisniewska DM, Johnson M, Beedholm K, Madsen PT (2012). Adaptive prey tracking by echolocating porpoises studied with acoustic tags. J. Acoust. Soc. Am..

[7] Linnenschmidt M, Teilmann J, Akamatsu T, Dietz R, Miller LA (2013). Biosonar, dive, and foraging activity of satellite tracked harbor porpoises (*Phocoena phocoena*). Mar. Mamm. Sci..

[8] Wisniewska DM (2016). Ultra-high foraging rates of harbor porpoises make them vulnerable to anthropogenic disturbance. Curr. Biol..

[9] Wahlberg M, Delgado-García L, Kristensen JH (2017). Precocious hearing in harbour porpoise neonates. J. Comp. Physiol..

[10] Kastelein RA, Hoek L, de Jong CAF, Wensveen PJ (2010). The effect of signal duration on the underwater detection thresholds of a harbor porpoise (*Phocoena phocoena*) for single frequency-modulated tonal signals between 0.25 and 160 Khz. J. Acoust. Soc. Am..

[11] Verfuß UK (2007). Geographical and seasonal variation of harbour porpoise (*Phocoena phocoena*) presence in the German Baltic Sea revealed by passive acoustic monitoring. J. Mar. Biol. Assoc. UK.

[12] Kyhn LA (2008). Harbour porpoise (Phocoena phocoena) static acoustic monitoring: laboratory detection thresholds of T-Pods are reflected in field sensitivity. J. Mar. Biol. Assoc. UK.

[13] Brandt MJ, Diederichs A, Betke K, Nehls G (2011). Responses of harbour porpoises to pile driving at the Horns Rev II offshore wind farm in the Danish North Sea. Mar. Ecol. Prog. Ser..

[14] Koblitz JC (2012). Asymmetry and dynamics of a narrow sonar beam in an echolocating harbor porpoise. J. Acoust. Soc. Am..

[15] Wisniewska, D. M. *et al.* Fast dynamic control over acoustic field of view by echolocating porpoises. *Elife* (2015). 10.7554/eLife.05651 PMC4413254 25793440

[16] Carstensen J, Henriksen OD, Teilmann J (2006). Impacts of offshore wind farm construction on harbour porpoises: acoustic monitoring of echolocation activity using porpoise detectors (T-Pods). Mar. Ecol. Prog. Ser..

[17] Tougaard J, Damsgaard Henriksen O, Miller LA (2009). Underwater noise from three types of offshore wind turbines: estimation of impact zones for harbor porpoises and harbor seals. J. Acoust. Soc. Am..

[18] Brandt MJ (2018). Disturbance of harbour porpoises during construction of the first seven offshore wind farms in Germany. Mar. Ecol. Prog. Ser..

[19] Graham Isla M (2019). Harbour porpoise responses to pile-driving diminish over time. R. Soc. Open Sci..

[20] Cox TM, Read AJ, Solow A, Tregenza N (2001). Will harbour porpoises (*Phocoena phocoena*) habituate to pingers?. J. Cetacean Res. Manage..

[21] Larsen F, Krog C, Eigaard OR (2013). Determining optimal pinger spacing for harbour porpoise bycatch mitigation. Endang. Species Res..

[22] Larsen F, Eigaard OR (2014). Acoustic alarms reduce bycatch of harbour porpoises in Danish North Sea gillnet fisheries. Fish. Res..

[23] Kyhn LA (2015). Pingers cause temporary habitat displacement in the harbour porpoise *Phocoena phocoena*. Mar. Ecol. Prog. Ser..

[24] Kindt-Larsen L, Berg CW, Northridge S, Larsen F (2019). Harbor porpoise (*Phocoena phocoena*) reactions to pingers. Mar. Mamm. Sci..

[25] Stedt, J. *et al.* Diurnal and lunar effects on acoustic detections of harbour porpoises (*Phocoena phocoena*) around Kullaberg, Sweden. In: Proceedings of the 29th annual conference of the European Cetacean Society, 2015 March 23–25, St Julian’s Bay, Malta. Abstract number ACO-16 (2015).

[26] Schaffeld T (2016). Diel and seasonal patterns in acoustic presence and foraging behaviour of free-ranging harbour porpoises. Mar. Ecol. Prog. Ser..

[27] Carlström J (2005). Diel variation in echolocation behavior of wild harbor porpoises. Mar. Mamm. Sci..

[28] Benjamins S, van Geel N, Hastie G, Elliott J, Wilson B (2017). Harbour porpoise distribution can vary at small spatiotemporal scales in energetic habitats. Deep Sea Res. Part II.

[29] Brandt MJ, Hansen S, Diederichs A, Nehls G (2014). Do man-made structures and water depth affect the diel rhythms in click recordings of harbor porpoises (*Phocoena phocoena*)?. Mar. Mamm. Sci..

[30] Cardinale M, Casini M, Arrhenius F, Håkansson N (2003). Diel spatial distribution and feeding activity of herring (*Clupea harengus*) and sprat (*Sprattus sprattus*) in the Baltic Sea. Aquat. Living Resour..

[31] Nilsson FLA (2003). Vertical migration and dispersion of sprat (*Sprattus sprattus*) and herring (*Clupea harengus*) schools at dusk in the Baltic Sea. Aquat. Living Resour..

[32] Neat FC (2006). Residency and depth movements of a coastal group of Atlantic cod (*Gadus morhua* L.). Mar. Biol..

[33] Benoit-Bird KJ, Dahood AD, Würsig B (2009). Using active acoustics to compare lunar effects on predator–prey behavior in two marine mammal species. Mar. Ecol. Prog. Ser..

[34] Grabowski TB, McAdam BJ, Thorsteinsson V, Marteinsdóttir G (2015). Evidence from data storage tags for the presence of lunar and semi-lunar behavioral cycles in spawning Atlantic cod. Environ. Biol. Fishes.

[35] Dähne M, Verfuß UK, Brandecker A, Siebert U, Benke H (2013). Methodology and results of calibration of tonal click detectors for small Odontocetes (C-Pods). J. Acoust. Soc. Am..

[36] Culik B, von Dorrien C, Müller V, Conrad M (2015). Synthetic communication signals influence wild harbour porpoise (*Phocoena phocoena*) behaviour. Bioacoustics.

[37] Scheidat M, Tougaard J, Brasseur S, Carstensen J, van Polanen Petel T, Teilmann J, Reijnders P (2011). Harbour porpoises (*Phocoena phocoena*) and wind farms: a case study in the Dutch North Sea. Environ. Res. Lett..

[38] Lazaridis, E. Lunar: lunar phase & distance, seasons and other environmental factors. *R package* version 0.1‐04 (2014).

[39] Thieurmel, B. & Elmarhraoui, A. Suncalc: compute sun position, sunlight phases, moon position and lunar phase. *R Package* version 50 (2019).

[40] Peres-Neto PR, Olden JD (2001). Assessing the robustness of randomization tests: examples from behavioural studies. Anim. Behav..

[41] Agostinelli, C. & Lund, U. R package Circular: circular statistics (version 0.4–93). CA: Department of Environmental Sciences, Informatics and Statistics, Ca’foscari University, Venice, Italy. UL: Department of Statistics, California Polytechnic State University, San Luis Obispo, California, USA (2017).

[42] Ruxton GD (2017). Testing for departure from uniformity and estimating mean direction for circular data. Biol. Let..

[43] R Core Team (2018). R: A Language and Environment for Statistical Computing.

[44] Akamatsu T, Hatakeyama Y, Kojima T, Soeda H, Kastelein RA, Supin AY, Thomas JA (1992). The rate with which a harbor porpoise uses echolocation at night. Marine Mammal Sensory Systems.

[45] Sørensen TB, Kinze CC (1994). Reproduction and reproductive seasonality in Danish harbour porpoises, Phocoena phocoena. Ophelia.

[46] Simonis AE (2017). Lunar cycles affect common dolphin Delphinus delphis foraging in the Southern California Bight. Mar. Ecol. Prog. Ser..

[47] Owen K, Andrews RD, Baird RW, Schorr GS, Webster DL (2019). Lunar cycles influence the diving behavior and habitat use of short-finned pilot whales around the main Hawaiian Islands. Mar. Ecol. Prog. Ser..

